# Healthcare-associated parechovirus-A infection in hospitals with neonatal units in Japan: a nationwide survey

**DOI:** 10.1017/ash.2026.10408

**Published:** 2026-06-29

**Authors:** Yuta Aizawa, Ichiro Morioka, Naoto Takahashi, Akihiko Saitoh

**Affiliations:** 1 Pediatrics, Niigata University Graduate School of Medical and Dental Sciences, Niigata, Japan; 2 Pediatrics and Child Health, Nihon University School of Medicine, Tokyo, Japan; 3 Pediatrics, The University of Tokyo, Tokyo, Japan

## Abstract

**Objective:**

To determine the scope and characteristics of healthcare-associated parechovirus-A infection (HA-PI).

**Design:**

Cross-sectional survey.

**Setting:**

Hospitals with neonatal units in Japan.

**Participants:**

Patients with parechovirus-A infection in 2023 in Japan.

**Methods:**

A 2-step questionnaire survey included the primary survey ascertaining the number of patients with HA-PI in 2023, followed by the secondary survey investigating the details of the patients with HA-PI.

**Results:**

Of the 408 hospitals, 226 (55.4%) responded. Seven sporadic patients were reported from 6 (2.7%) hospitals between May and September 2023, diagnosed as having sepsis (n = 5) or sepsis-like illness (n = 2). The median gestational age was 37.7 weeks (IQR, 36.2–38.3). They were second-born (n = 6) or third-born (n = 1), without first-born. The median days of onset of illness were 7 days (IQR, 6–15). The locations were the newborn nursery (n = 3), growth care (n = 3), or neonatal intensive care units (n = 1). No sequelae were found at least 10 months in all patients. Parechovirus-A genotype analyses demonstrated A3 (n = 6) and unknown (n = 1). The identified sources of infection were from the mother (n = 5) or unknown (n = 2). Among the 5 mothers, 2 were symptomatic. Notably, the siblings of patients in 3 asymptomatic mothers, who had no direct contact with the patients, were all symptomatic.

**Conclusions:**

This nationwide survey in Japan demonstrated HA-PI can occur in neonatal units, with potential risks for nosocomial outbreaks. Suspecting HA-PI and preventive measures are critical in neonatal units.

## Introduction

Parechovirus-A (PeV-A), formerly named human parechovirus, are RNA viruses of the genus *Parechovirus* in the family *Picornaviridae*.^1^ Generally, PeV-A infection causes mild upper respiratory or gastrointestinal symptoms, ranging from asymptomatic to severe infection.^2^ Among 19 genotypes classified by viral protein 1 sequences, PeV-A genotype 3 (PeV-A3) is a major cause of viral sepsis and encephalitis in neonates and young infants aged <4 months. PeV-A3-related severe diseases in infants are well-known as community-acquired infections.^3^

Recently, we experienced an outbreak of PeV-A3 infection in a newborn nursery in Japan.^4^ To date, there have been only 2 reports of healthcare-associated PeV-A infection (HA-PI), which were single-center studies of outbreaks in a maternity unit in Austria and Hungary.^5,6^ Given that PeV-A3 causes severe diseases in neonates, a healthcare-associated outbreak poses a substantial risk of significant consequences for both the affected neonates, family, and the healthcare facility. However, even the reported frequency of symptomatic viral respiratory infections in the neonatal intensive care unit (NICU), which ranges from 1% to 8%,^7,8^ is likely an underestimate. This is attributed to the fact that respiratory viruses are not routinely tested for, given the seemingly low incidence of respiratory viral infections within the NICU setting.^9^ Furthermore, it is even more challenging to suspect a viral infection based solely on signs such as sepsis-like syndrome or apneic episodes,^10^ as these manifestations can be caused by other factors, including bacterial infections, hypoglycemia, and intracranial hemorrhage. Importantly, these symptoms are also typical manifestations of neonatal PeV-A infection.^10^

Therefore, it is crucial to accurately determine the scope and characteristics of HA-PI. However, the actual incidence of HA-PI remains insufficiently characterized. To address this gap, we conducted a nationwide survey on HA-PI in 2023, when there was an increase in the number of community-acquired PeV-A3 infection in neonates and young infants in Japan following the relaxation of the coronavirus disease 2019 restrictions.^11^

## Methods

A 2-step questionnaire survey was conducted in collaboration with the Japan Society for Neonatal Health and Development.

### Phase 1 survey

The phase 1 survey was conducted with the dual objectives of ascertaining the number of patients with HA-PI in 2023 and assessing the foundational diagnostic capacity and opportunities for diagnosing PeV-A infection at each hospital. The latter objective was set because it remains unclear whether HA-PI in NICUs is underreported or truly rare events, and because the information on preparedness for HA-PI in NICUs is crucial to interpret the incidence of HA-PI in this study. In Japan, a multiplex polymerase chain reaction (PCR) assay including PeV-A for cerebrospinal fluid (CSF) samples was not widely adopted before the coronavirus disease 2019 (COVID-19) pandemic; however, the COVID-19 pandemic triggered a rapid increase in its implementation in actual clinical settings following the introduction of a multiplex PCR assay for respiratory samples.^11^ In April 2024, web-based questionnaires were sent to the NICU supervising physicians or directors of the pediatric department of 408 certified perinatal medical centers nationwide, which were registered and publicly announced by the Ministry of Health, Labour and Welfare of Japan as of July 14, 2023, and providing neonatal care. Three hospitals were excluded where adjacent hospitals provide neonatal care, and the hospital only provides care for pregnant women. The questions included (1) whether screening for viral pathogens is performed when an infection is suspected in a patient admitted to or currently hospitalized in the NICU, growth care unit (GCU), or newborn nursery, and bacterial or fungal infection is ruled out by the culture, (2) the testing methods used when PeV-A infection is suspected (multiple responses and open-ended comments allowed), (3) whether the hospital had experience in treating patients who were laboratory-diagnosed as having PeV-A infection among neonates in newborn nursery or patients in the NICU or GCU (including transfers from other maternity hospitals) in 2023. The phase 1 survey was completed in May 2024.

### Phase 2 survey

Hospitals that experienced HA-PI cases in 2023 were asked to provide detailed information via e-mail. The following information was collected using electronic health record system; the number of PeV-A infection cases treated in 2023, clinical information for each case (month of admission, gestational age, birth weight, onset day of illness, presumed location of infection, presumed source of infection, symptoms of the presumed source of infection, symptoms in the patient with PeV-A infection, presence or absence of sequelae), and laboratory information for each case (results of blood and CSF tests, availability of brain magnetic resonance imaging (MRI) and its results if available, PeV-A testing method, type of sample PeV-A was detected, and PeV-A genotype). The phase 2 survey was completed in November 2024.

This study was approved by the Ethics Committee of Niigata University (2023-0319) and administrative board of Japan Society for Neonatal Health and Development.

### Definitions

HA-PI was defined as a disease onset after birth, but before the hospital discharge, with PCR-confirmed PeV-A infection by serum or CSF samples, which was a definitive diagnosis of systemic or central nervous system infection of PeV-A.^12^ Sepsis was defined using the definitions of the international pediatric sepsis consensus conference.^13^ Sepsis-like illness was defined as a disease that did not meet the criteria for sepsis but the patient presented with poor general appearance, or abnormal physical findings, such as mottled skin. Duration of the disease was defined as the period from the day of initial deviation from the baseline condition to the day of recovery to baseline.

### Infection prevention measures in neonatal units in Japan

Although infection prevention measures in NICUs and GCUs varied across Japan,^14^ strict adherence to universal personal protection equipment (gowns, gloves, and face masks) in addition to hand hygiene has been established. Infection prevention measures in neonatal units other than NICUs and GCUs are thought to be highly diverse depending on institution-specific policies, which was outside the scope of the current study. Visitors were screened routinely for cold symptoms and contacts of contagious diseases before meeting their infants in neonatal units. Sibling visitation was permitted in only a limited number of NICUs even before the COVID-19 pandemic,^15^ and it remained uncommon in the postpandemic era.

## Results

### Phase 1 survey

Of the 408 hospitals, 226 (55.4%) hospitals responded. Viral testing is conducted on a case-by-case basis in 182 (80.5%) hospitals and universally in 22 (9.7%) hospitals, although principally not conducted in 22 (9.7%) hospitals.

PeV-A was tested using the BioFire Filmarray meningitis/encephalitis panel at their own laboratories in 96 (42.5%) hospitals, sending samples to the local health laboratories in 96 (42.5%) hospitals or to the university/other hospitals available for PeV-A testing in 52 (23.0%) hospitals, using in-house PCR other than the BioFire Filmarray meningitis/encephalitis panel in 20 (8.8%) hospitals, and sending samples to the commercial laboratories only available for virus isolation in 3 (1.3%) hospitals. Twenty (8.8%) hospitals only reported the unavailability of PeV-A testing at their own laboratories without indicating any alternative testing methods, and 4 (1.8%) hospitals reported that they had never even suspected PeV-A infection.

In 2023, a total of 7 patients with HA-PI were reported from 6 (2.7%) hospitals.

### Phase 2 survey

**Figure 1.**
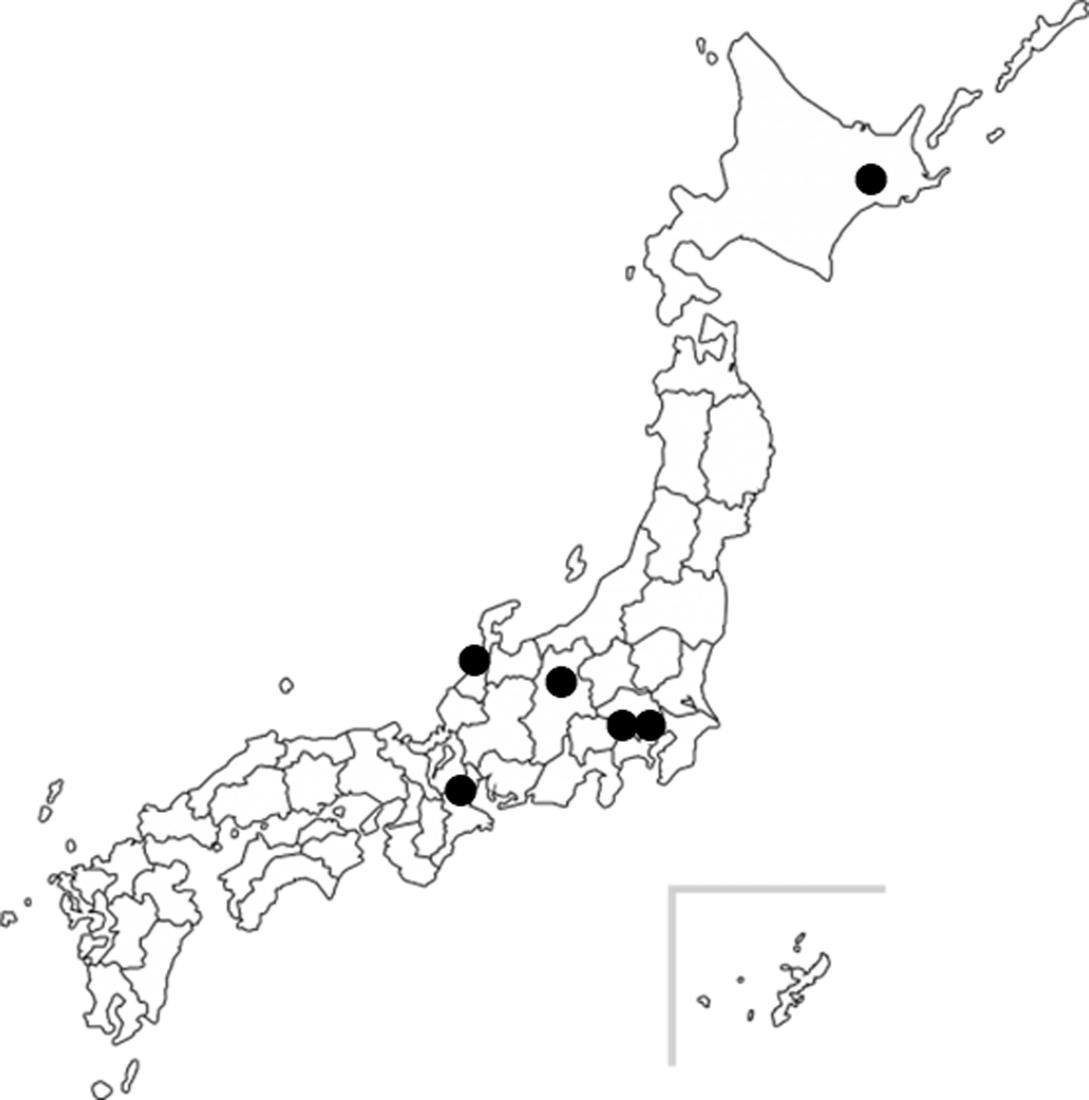
Six hospitals with neonatal units that reported patients with healthcare-associated parechovirus-A infection in 2023.

The 6 reporting hospitals were geographically distributed without apparent bias (Figure 1). The patients with HA-PI were identified between May and September (Table 1). Three patients (Patients 3, 4, and 5) were transferred from nearby maternity hospitals due to clinical deterioration. No clusters of HA-PI were observed at any hospital; the 2 patients reported by one hospital occurred 3 months apart.

**Table 1.** Details of 7 patients with healthcare-associated parechovirus-A infection

	Patient 1	Patient 2	Patient 3	Patient 4	Patient 5	Patient 6	Patient 7
Month of HA-PI	May	May	June	July	August	August	September
Gestational age, weeks and days	36w0d	36w3d	38w1d	35w6d	38w3d	37w5d	38w5d
Birth weight, grams	2,064	2,505	2,480	2,738	2,314	3,020	2,874
Sex	Female	Female	Female	Female	Female	Male	Female
Perinatal complications	Preterm LBI	Preterm Laryngomalacia	LBI	Preterm	LBI	TTN	None
Birth order	Second-born	Second-born	Second-born	Third-born	Second-born	Second-born	Second-born
Onset of illness, days	15	54	6	5	7	15	6
Place of occurrence	NICU	GCU	Newborn nursery	Newborn nursery	Newborn nursery	GCU	GCU
Symptoms							
Lethargy	+	+	+	+	+	+	+
Poor sucking	+	−	+	+	+	−	+
Fussiness	−	+	+	−	+	+	+
Apnea	+	−	−	+	+	−	+
Rash	–	+	−	+	−	+	+
Vomiting	−	−	−	+	−	−	+
Convulsion	−	−	−	−	−	−	+
Peak body temperature, °C	37.6	39.0	39.0	37.8	38.5	38.4	38.6
Signs							
Abdominal distention	+	+	+	+	−	+	+
Cold extremities	−	+	+	+	+	+	+
Mottled skin	−	+	−	+	+	+	+
Irritability	−	+	+	−	−	+	+
Palmar-plantar erythema	−	+	−	+	−	+	+
Delayed CRT (>2 s)	−	−	−	+	+	−	−
Umbilical protrusion	−	+	−	−	−	−	+
Laboratory findings							
White blood cells, /μL	2,500	8,000	10,600	6,430	3,960	4,700	4,380
AST, IU/L	25	91	34	34	42	32	35
ALT, IU/L	11	35	13	9	21	11	15
LDH, IU/L	221	491	482	436	273	456	475
CRP, mg/dL	.00	.01	.05	.09	.05	.01	.08
CSF cell count, /μL	15	N.A.	3	<1	3	1	1
CSF glucose, mg/dL	53	N.A.	51	115	59	53	55
CSF protein, mg/dL	120	N.A.	53	67	96	64	76
Diagnosis	Sepsis	Sepsis	Sepsis	Sepsis-like illness	Sepsis	Sepsis	Sepsis-like illness
Brain MRI findings^a^	Abnormal	Normal	N.A.	Abnormal	N.A.	Normal	Abnormal
Illness duration, days	5	5	5	7	8	6	8
Site of PeV-A detection^b^	Serum	Serum	Serum, CSF nasopharyngeal swab	Serum CSF	CSF	CSF	CSF
PeV-A genotype	3	3	3	3	Unknown	3	3

Note. AST, aspartate aminotransferase; ALT, alanine aminotransferase; CRP, C-reactive protein; CRT, capillary refill time; CSF, cerebrospinal fluid; GCU, growth care unit; HA-PI, healthcare-associated parechovirus-A infection; LBI, low birth weight; LDH, lactate dehydrogenase; MRI, magnetic resonance imaging; N.A., not available; NICU, neonatal intensive care unit; PeV-A, parechovirus-A; TTN, transient tachypnea of newborn.

No one had sequelae at least 10 months of age.

^a^Abnormal brain MRI findings indicated bilaterally symmetric high signal intensity on diffusion-weighted imaging in the dorsal aspect of the corpus callosum and the peritrigonal walls of the lateral ventricles, accompanied by T1 high signal and T2 low signal intensity in the cerebral white matter (Patient 1); scattered high signal intensity foci in the cerebral white matter on fluid attenuated inversion recovery and T1-weighted images (Patient 4); multiple areas of restricted diffusion in the bilateral deep cerebral white matter (Patient 7).

^b^All sample types submitted for PeV-A detection were not asked in the survey.

#### Patients with HA-PI

The details of the 7 patients with HA-PI are summarized (Table 1). Preterm infants included 3 (42.9%) patients, and 6 (85.7%) patients were female. The 6 (85.7%) patients were hospitalized due to perinatal complications. No cases were reported in first-born infants, and the highest number of cases was recorded in those who were second-born (n = 6, 85.7%). The median of illness onset was 7 days of age (interquartile range [IQR], 6–15 days). At the onset of symptoms, the patients were in the newborn nursery from nearby maternity hospital (n = 3, 42.9%), GCU (n = 3, 42.9%), and NICU (n = 1, 14.3%). All patients were lethargic, and 5 (71.4%) patients were febrile (>38°C). Four (57.1%) patients showed rash and apnea, and 1 (14.3%) patient had convulsion. No one had symptoms of cough, rhinorrhea, or diarrhea. Cold extremities and abdominal distension were the most frequently observed signs (n = 6, 85.7% for each). Laboratory findings were unremarkable, showing no elevation in inflammatory markers or CSF pleocytosis. The patients were diagnosed as having sepsis (n = 5, 71.4%) or sepsis-like illness (n = 2, 28.6%).

Antibiotics were administered in all patients, although acyclovir was given in 3 (42.9%) patients. No one required catecholamines. Respiratory support was required for 4 (57.1%) patients via non-invasive positive pressure ventilation and for 1 (14.3%) patient via invasive mechanical ventilation. Abnormal findings in brain MRI were observed in 3 of 5 patients tested. The median duration of illness was 6 days (IQR, 5–7.5 days), and no one had sequelae at least 10 months of age.

The patients were diagnosed as having PeV-A infection using the BioFire Filmarray meningitis/encephalitis panel (n = 4, 57.1%), at the laboratory of university or hospital (n = 2, 28.6%), and at the local health laboratory (n = 1, 14.3%). Subsequently, the viruses were genotyped as PeV-A3 (n = 6, 85.7%) and unknown (n = 1, 14.3%).

#### Source of infection and context

Of the 7 patients, the source of infection was attributed to the mother in 5 (71.4%) cases, with the source being unknown in 2 (28.6%) cases (Table 2).

**Table 2.** Source of infection and context in 7 patients with healthcare-associated parechovirus-A infection

	Patient 1	Patient 2	Patient 3	Patient 4	Patient 5	Patient 6	Patient 7
Source of infection	Unknown	Mother	Unknown	Mother	Mother	Mother	Mother
Symptoms of mothers	N.A.	None	N.A.	Arthralgia Lethargy	None	None	Diarrhea
Symptoms of siblings	N.A.	Vomiting	N.A.	None	Diarrhea Fever	Rhinorrhea	N.A.

Note. N.A., not available.

Of the 2 symptomatic mothers, 1 mother in Patient 4 presented with arthralgia and lethargy, and the siblings of Patient 4 were asymptomatic. The other mother in Patient 7 reported diarrhea. Although the symptomatic status of the sibling of Patient 7 was unknown, sibling visitation to neonate and mother was not permitted.

Siblings were all symptomatic in 3 asymptomatic mothers, although siblings had no direct contact with the patients. Of the 3 asymptomatic mothers, 1 was visited by the 3-year-old sister after the child had recovered from vomiting (Patient 2). Another mother was in contact with the 2-year-old brother, who subsequently developed diarrhea and fever (Patient 5). The remaining mother was visited by the 2-year-old brother following recovery from rhinorrhea (Patient 6).

In 1 of the 2 cases in which the source of infection was not identified, a healthcare staff member with cold symptoms was identified (Patient 1).

## Discussion

This is the first study to investigate PeV-A infection at the national level from the perspective of healthcare-associated infection (HAI). This multicenter study in Japan revealed that PeV-A infection causes HAIs in neonatal units, not limited to community-acquired infection.

The clinical manifestations and PeV-A genotype in the current study were consistent with those previously reported in the literature.^4–6^ In these previous reports, the neonates with HA-PI presented with sepsis-like illness or fever and rash, and all cases where the genotype was identified were uniformly PeV-A3. All patients in the previous reports also recovered without sequelae. It is crucial to consider HA-PI as a differential diagnosis in infants with sepsis or sepsis-like illness in the neonatal units, with or without a rash. Simultaneously, it is essential to ensure preparedness for testing by establishing in-hospital PCR capabilities or collaborating with external laboratories.

For the management of HAI, the identification of the source of infection and its subsequent transmission route is essential. For infants in the NICU to contract a respiratory virus, transmission typically occurs through direct deposition from visitors or healthcare workers, or via contact with contaminated hands and shared fomites.^9^ Similar transmission routes are also plausible for PeV-A, which primarily replicates in the gastrointestinal tract and is mainly transmitted through fecal-oral route.^2^ Horizontal transmission route was strongly suspected in the current study. The median (IQR) of illness onset was 7 (6–15) days beyond the incubation period of PeV-A3 infection, 1–3 days (at maximum, 7 days),^4^ making vertical transmission unlikely. Furthermore, even asymptomatic mothers had symptomatic siblings. It is assumed that symptomatic siblings transmitted PeV-A3 to the mother during a visit to her hospital room or at home, and even asymptomatic mother transmitted PeV-A3 to neonates in newborn nursery or patients in the NICU or GCU, because asymptomatic person can transmit PeV-A3 to the neonates and young infants.^16,17^

In previous reports concerning HA-PI, the sources of infection and transmission routes were diverse. A report from Hungary indicated that a family member with respiratory symptoms visited the mother postdelivery. Although the mother was asymptomatic, PeV-A3 was transmitted from the mother to the neonate, subsequently spreading to 4 other neonates in a maternal unit. Direct deposition or airborne transmission, rather than direct contact transmission, was hypothesized as the primary route of spread.^6^ A report from Austria noted that both the source and the transmission route of the PeV-A3 infection remained unidentified. However, given the absence of direct contact between the affected neonates, an asymptomatic adult or a sibling was hypothesized to be the common source of infection.^5^ A report from Japan speculated that PeV-A3 was introduced by an asymptomatic pregnant woman and subsequently transmitted to the neonate via vertical transmission. The subsequent spread was deemed most likely to be contact transmission mediated by mothers’ or healthcare workers’ hands or environmental surfaces. This conclusion was based on 2 factors: temporary overcrowding in the newborn nursery and non-adherence to routine cleaning protocols for shared items (eg, bathing tubs, scales) and high-touch surfaces (eg, doorknobs, railings).^4^ Given the findings of this study and previous reports, strict hand hygiene before touching an infant is essential when there has been exposure to individuals with cold symptoms, even if the caregiver is asymptomatic. Furthermore, wearing a mask should be considered when caring for high-risk infants.

This study has a few limitations. First, the number of HA-PI might have been underestimated given the response rate of the primary survey was approximately 50%. Second, the samples of the mothers and siblings were not available for PCR testing due to the nature of the retrospective study, which precluded the verification of the PeV-A3 shedding by the mothers and siblings. It would help better understand possible transmission routes. Last, only 7 patients with HA-PI were observed during a year with a relatively high number of PeV-A3 infections in the community.^11^ The generalizability of the rate of HA-PI to other years, especially when PeV-A3 is endemic, is uncertain. Thus, it is warranted to continue monitoring of HA-PI.

In conclusion, HA-PI were reported from 2.7% of hospitals with neonatal units in Japan. HA-PI can occur in neonatal units, with potential risks for nosocomial outbreaks. Suspecting HA-PI and preventive measures are critical in neonatal units.

## Supporting information

10.1017/ash.2026.10408.sm001 Aizawa et al. supplementary material Aizawa et al. supplementary material

## Data Availability

The data are available upon reasonable request.

## Supplementary material

The supplementary material for this article can be found at https://doi.org/10.1017/ash.2026.10408.
